# The effects of cognitive-motor training interventions on executive functions in older people: a systematic review and meta-analysis

**DOI:** 10.1186/s11556-020-00240-y

**Published:** 2020-07-02

**Authors:** Bettina Wollesen, Alicia Wildbredt, Kimberley S. van Schooten, Mei Ling Lim, Kim Delbaere

**Affiliations:** 1 Department of Human Movement Science, University of Hamburg, Hamburg, Germany; 2 Biological Psychology and Neuroergonomics, Technical University of Berlin, Fasanenstr. 1, D-10623 Berlin, Germany; 3 Neuroscience Research Australia, Sydney, New South Wales Australia; 4 School of Public Health and Community Medicine, Faculty of Medicine, University of New South Wales, Sydney, New South Wales Australia

**Keywords:** Aged_1_, Dual-task_2_, Exergame_3_, Motor-cognitive intervention_4_, Cognitive function_5_

## Abstract

**Background:**

Ageing is associated with physical and cognitive decline, affecting independence and quality of life in older people. Recent studies show that in particular executive functions are important for daily-life function and mobility. This systematic review investigated the effectiveness of cognitive-motor training including exergaming on executive function (EF, set-shifting, working memory, inhibitory control) in healthy older people.

**Methods:**

An electronic database search for randomised controlled trials (RCT), controlled clinical trials (CCT) and parallel group trials was performed using MEDLINE, EMBASE, and PsychINFO following PRISMA guidelines. Inclusion criteria were: (1) community-dwelling participants > 60 years without a medical condition or medical treatment, (2) reporting at least one cognitive-motor intervention while standing or walking, (3) use of dual-task interventions using traditional methods or modern technology to deliver a cognitive-motor task, (4) inclusion of at least one cognitive outcome. The PEDro scale was used for quality assessment.

**Results:**

A total of 1557 studies were retrieved, of which 25 studies were included in this review. Eleven studies used a technology-based dual-task intervention, while 14 trials conducted a general cognitive-motor training. The age range of the cohort was 69 to 87 years. The interventions demonstrated positive effects on global cognitive function [mean difference 0.6, 95% CI 0.29–0.90] and inhibitory control [mean difference 0.61, 95% CI 0.28–0.94]. Effects were heterogeneous (I^2^ range: 60–95) and did not remain after a sensitivity analysis. Processing speed and dual-task costs also improved, but meta-analysis was not possible.

**Conclusion:**

Cognitive-motor and technology-based interventions had a positive impact on some cognitive functions. Dual-task interventions led to improvements of domains related global cognitive functions and inhibitory control. Likewise, technology-based exergame interventions improved functions related to processing speed, attentional and inhibitory control. Training interventions with a certain level of exercise load such as progression in difficulty and task specificity were more effective to gain task-related adaptations on cognitive functions.

## Introduction

Ageing is associated with changes to the locomotor system and reduced efficiency of cognitive processing [1]. Most daily activities require the simultaneous execution of cognitive and motor tasks [2]. Limited resources, reduced efficiency and increased interference between tasks often lead to deterioration of motor and/or cognitive performance with ageing [3, 4]. Reduced performance while performing a simultaneous task, often referred to as cognitive-motor interference, represents a major risk factor of falls in older people [5–8]. Therefore, adequate physical and cognitive resources with the ability to use these resources simultaneously seems key in order for older people to maintain an independent lifestyle.

Executive functions (EF) are the main cognitive abilities necessary to interact with the environment in daily activities [9]. Executive functions include a wide range of higher-order cognitive functions. A recent review by Diamond [10] identified three major domains of EF that are of particular relevance to walking and daily life activities [11–13]: (i) inhibitory control, which includes the ability to stay focused despite distraction and inhibit pre-potent but inappropriate responses; (ii) working memory, which refers to the ability to hold and manipulate information, prioritise and plan actions, and (iii) cognitive flexibility, which refers to one’s ability to adjust and change attention, as well as set-shifting and task-switching (cf. Textbox 1).

**Table:** 

**Textbox 1- Cognition and executive function taxonomy** The term “cognition” is a broad expression of mental domains related to brain processes connected to assimilating and understanding external stimulation [14] and “the ability to hold, process and manipulate information in the mind” ([15], p.5). One major important set of cognitive processing is described by the term “executive function” (EF), which is also referred to as cognitive control [10]. According to the definition of Diamond [10], EF is an umbrella term for a collection of mental processes regarding the ability to concentrate, focus and adequately react to external stimuli. Based on this it is of general agreement that EFs are subdivided into three core elements *inhibition, working memory* and *cognitive flexibility* [10]. 1. Inhibition describes the ability to control attentional resources, e.g. connected to behaviour, thoughts or emotions. Inhibition enables an individual to selectively focus on an external stimulus to be processed, while suppressing other stimuli. ° Inhibitory control is often measured by testing one’s selective attention which requires the ability to correctly discriminate two incongruent stimuli, e.g. the Stroop, the Flanker Test or tasks involving a go/no-go instruction. 2. Working memory (WM) involves holding and manipulating information in mind, e.g. using stored information to solve an ongoing problem. Linked to a variety of neuronal subsystems, WM enables the analysis and clustering of information while selectively focusing on information stored in mind. Testing WM often requires reordering stored information, e.g. repeat a selection of numbers in another order, which is for example used in complex span-backwards tasks, e.g. the n-back task. ° Verbal fluency tasks require the ability to hold information in mind, while performing a mental process, e.g. the spontaneous production of words. ° Arithmetic tasks refer to tasks solving mathematical problems, e.g. counting backwards or multiplying. 3. Cognitive flexibility describes the ability to change perspectives according to external demands and an adequate reaction. Flexibility is often investigated via task switching (set-shifting) tasks. This requires the ability to randomly shift between various required stimulus-respond set, e.g. during the Wisconsin Sorting Card test [15]. Other basic cognitive functions regarding *reaction time* and speed of cognitive processing [16]: 1. Processing speed is referred to as the simple reaction time between an external stimulus and a behavioural response occurrence, which can be tested by the finger-tapping speed as a reaction of a visual stimulus. 2. Visuospatial abilities are connected to the processing and memory of visual as well as spatial stimuli. Common test for visuospatial planning abilities are the Clock-Drawing-Test or the Spatial Span test.	

Previous research has shown that it is possible to improve cognitive function in older people, using physical and mental training, individually or combined. Combining physical and mental training is often referred to as dual-task training (DT) [8, 17, 18]. Dual-tasking describes the combination of a motor and a cognitive task; e.g. walking while counting backwards, conducting a verbal fluency task (e.g. naming animals), attentional control (e.g. reaction on signs) and processing speed [8]. Generally, simultaneous demands can lead to a reduced performance in one of the two tasks. The change in performance under dual-task compared to single-task conditions is called a dual-task cost [7], otherwise known as cognitive-motor interference [19]. In a recent review, Tait and colleagues [18] demonstrated that training motor and cognitive tasks simultaneously (dual-task training) showed significant improvements in cognitive functions, i.e. global cognition and EF (inhibition, working memory and cognitive flexibility), compared to sequential physical and mental training.

Recent studies have repeatedly shown that the ageing brain and body remain plastic and that older people’s performance can be improved through systematic motor or cognitive training [20–22]. However, improvements in neurophysiological correlates differ with the type of the training tasks [21]. Thus, through physical training, cognitive resources can be applied more effectively. Moreover, different types of dual- or multi-task training can positively influence cognitive performance of older people [8, 21]. While there are positive results, more work is required to uncover which types of dual-task training are most effective. Wollesen and Voelcker-Rehage [8] subdivided dual-task training into categories based on the training and assessment method, differentiating general and specific dual-task training. General dual-tasking (GDT) describes an intervention which uses a variety of dual-task exercises with the idea that this intervention might improve general dual-task performance. Specific dual-tasking (SDT) requires the participant to specifically conduct the same tasks as in the training condition. In line with these classifications, a General Dual-Task (GDT) program describes an intervention which uses different unspecific training tasks in comparison to the assessment tasks; while a Specific Dual-Task (SDT) program consists of training tasks that address a comparable cognitive dimension as the assessment task (cf. Textbox 2).

**Table:** 

**Textbox 2 – Classification of the cognitive-motor intervention** Cognitive-motor interventions are also referred to as dual-task (DT) interventions, which require the simultaneous conduction of a mental and a physical task, e.g. walking while counting backwards [8]. Wollesen and Voelcker-Rehage [8] subdivided dual-tasking into different categories in regard to the comparison of training and assessment method: • A *General dual-task training* (GDT) describes an intervention which uses different unspecific training tasks in comparison to the assessment tasks • During *Specific dual-task training* (SDT) the intervention consists of training tasks that addresses a comparable cognitive dimension as the assessment task In line with these classifications • A *General dual-task exergame* (GDT-EX) describes an exergame intervention which uses different unspecific training tasks in comparison to the assessment tasks A *Specific dual-task exergame* (SDT-EX) intervention consists of training tasks that address a comparable cognitive dimension as the assessment task	

Dual-task training programs have traditionally been delivered face-to-face in group settings or one-on-one. Positive effects on cognitive-motor performance can be observed if the training has a minimum duration of twelve sessions or 330 min as well as an advancement in task difficulty and progression [8]. Due to rapidly changing technologies, it has become more popular to utilise exercised-based computer games, also known as exergames, for dual-task training. If well-designed, exergames can add a high cognitive demand to physical training, possibly even making use of a virtual environment [19]. Exergaming might also have the benefit of being implemented as an unsupervised, home-based program, which can potentially reduce costs for users.

The purpose of this review was twofold: (i) to summarise existing studies using cognitive-motor interventions and their effects on global cognitive functioning as well as the three dimensions of EF (inhibitory control, working memory and cognitive flexibility) in older people aged 60 years and above, and (ii) to identify the common characteristics of effective interventions in terms of duration, frequency and task progression. We hypothesise that EF can be improved by a specific DT intervention including at least a duration of 330 min or twelve sessions and a progression of task difficulty and task complexity.

## Methods

The protocol for this review was prospectively registered with the International Prospective Register of Systematic Reviews (PROSPERO) with the registration number CRD 42018111083.

### Search strategy and selection criteria

A systematic electronic database search for relevant articles was performed using MEDLINE (1966 to 2019, week 40), EMBASE (1966 to 2019, week 40) and PsychINFO (1980 to 2019, week 40). The literature search included keywords related to older people, cognitive-motor as well as technology-based or exergaming interventions, and a cognitive outcome (cf. Table 1 for more details; a detailed list of the key terms is added as an Appendix). The process also included a manual search of the reference lists of relevant articles to identify articles that did not show up in the search strategy results. The search within the databases was limited to articles published in English. Two reviewers (AW and BW) independently reviewed the articles by title and abstract to identify all potentially eligible articles following the PRISMA methodology [23]. The process integrated the screening of the articles by title and abstract. All relevant articles regarding title and abstract were integrated into the full text screening. If the screening of the abstract did not clarify the inclusion or exclusion criteria, the full text was extracted. Afterwards, the two reviewers independently assessed full version copies of all potentially eligible articles to determine the ones to be included. Any disagreement on inclusion was resolved by discussion and through consensus by the larger project team (including KvS, ML and KD).

The study selection was limited to randomised controlled trials, control group designs trials and parallel group trials. Study protocols, abstracts or conference abstracts were excluded due to limited information. Articles were included if they met the methodological inclusion and exclusion criteria.

The target population included community-dwelling participants with a minimum age of 60 and a mean age above 65 without a medical condition or recent medical treatment. This included participants who lived independently in private homes, in independent-living units or retirement villages. We included participants with no cognitive deficits or with a diagnosis of mild cognitive impairment only. Therefore, we agreed to exclude studies including participants with pre-intervention scores below 25 on the MMSE or equivalent on the Montreal Cognitive Assessment (MoCA) score. Trials in which participants were selected based on a medical condition (e.g. stroke, Parkinson disease, multiple sclerosis, schizophrenia or dementia) and trials including only participants who received a recent medical treatment (e.g. surgery or medication) were excluded.

Trials with interventions using at least one cognitive-motor exercise while standing or walking were included. The training could be delivered using traditional dual-tasking methods or modern technology. Dual-tasking or cognitive-motor interventions required simultaneous cognitive and motor exercise. Additionally, interventions using technologies could use consoles, virtual reality systems or bespoke software. Trials presenting on seated training programs were excluded. We extracted whether the motor task was conducted while standing or walking due to the different levels of complexity of the motor tasks. When the intervention required the participant to work with a visual feedback or biofeedback as part of the technology-based game, this was considered a cognitive load.

Finally, each study had at least one cognitive outcome, evaluated as a primary or secondary outcome. These cognitive domains included general cognition, general executive function, set-shifting, inhibitory control, working memory, visuospatial planning, verbal fluency, attentional control, processing speed and dual-task costs.

### Data extraction and quality assessment

Eligible articles were screened independently by two review authors (AW, BW). Data on sample characteristics, type of a traditional motor-cognitive or technological exergaming intervention as well the frequency and duration of the intervention was extracted. Table 1 provides an overview of all included articles including the authors, year of publication, included participants, study aims, intervention, cognitive and motor task measurement, and training duration.

To assess risk of bias and quality, we used the PEDro scale [24]. Articles scoring 6–10 points on the PEDro quality assessment were considered to be of high quality, articles with a score of 4–5 of fair quality and articles with a score of 0–3 of poor quality (cf. Table 2).

### Statistical analysis of the meta-analysis

We extracted data of the intervention and control group for each of the outcome variables of interest (cf. Textbox 1) as a difference in means (MD). Most of the studies reported means and Standard Deviations (SD) permitting effect size estimation, otherwise, these were derived from summary statistics reported in the articles, such as *t*-values or *p*-values. The cognitive data from individual studies were then pooled in meta-analyses to estimate the overall effect of the training intervention on cognition. Studies were grouped by cognitive task domain and individual meta-analyses were conducted for each EF outcome. As the studies used different control groups (e.g. active controls with another intervention, for example a single-task motor intervention or an education program or inactive controls with normal daily activities cf. Table 3), the meta-analysis was conducted with a subgroup analysis regarding active and inactive controls. A random-effects model with a generic inverse variance method was used, which gives more weight to studies with less variance in the pooled analyses. Results are presented as effect size with 95% confidence interval (CI) and respective values for null hypothesis tests (e.g. cognitive-motor training has no effect on EF). Heterogeneity between studies was investigated by calculating the Q-value and I^2^ statistic, which quantified the proportion variation that is due to heterogeneity rather than chance. Quantitative syntheses and meta-analyses were produced using Review Manager 5 Software (RevMan 5).

## Results

### Classification of included studies

**Fig. 1:**
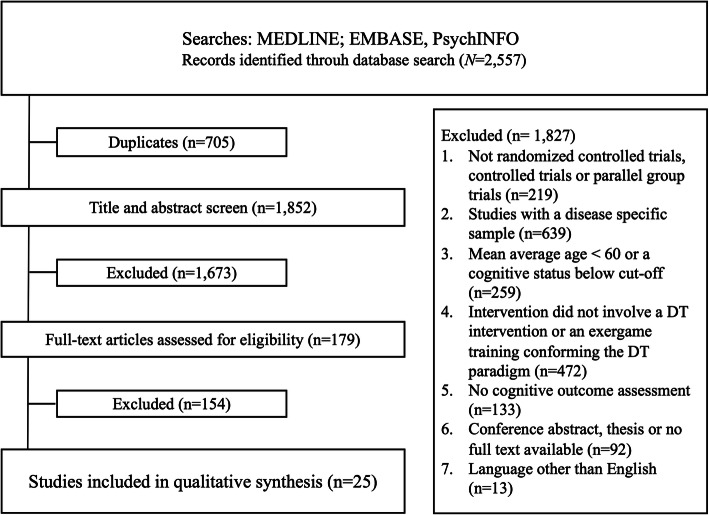
Flow chart of study selection process

The initial search generated 2593 articles of which 705 were duplicates (Fig. 1). For the final review, 25 articles were identified eligible and integrated into further assessment.

### Cohort characteristics

Studies included samples of 13 to 134 participants (median 36). The mean age of participants ranged from 66.8 to 86.0 years (cf. Table 1). Both genders were included in 18 of the trials, generally with a higher percentage of female participants. Seven studies did not provide any information on the gender of their participants [25–31].

**Table 1:** Characteristics of included studies

Eligible author and year	Participants (age(M,SD), sex)	Study aims	Intervention (cognitive-motor task)	Cognitive task and measurement	Motor task and measurement	Training duration
**Randomised controlled trials**						
Azadian (2016) [26]	*N* = 30 IG1:*n* = 10, 73.9 ± 5.5, n.a. IG2:*n* = 10, 73.8 ± 3.9, n.a. CG:*n* = 10, 73.7 ± 4.4; n.a.	evaluation of the effect of two cognitive training methods on pattern of gait	IG1: GDT IG2: CT CG: -	**INH/PS** (A-mo/Vi-mo): Reaction time to auditory stimulus/visual stimulus on screen; **WM:** Wechsler Adult Intelligence Scale, Digit Symbol B/F	walking ST and DT condition: Vicon System, asymmetry index (AS)	18 h/ 3 × 45 min p.w. for 8 wk.
Bacha (2018) [32]	*N* = 46 IG: *n* = 23, 8 m/15f CG: *n* = 23, 4 m/19f	effectiveness of KAG games versus CPT to improve postural control, gait, fitness and cognition	IG1: EXG CG: PHY	**GF:** MoCa	Postural Control: Mini-BESTest; Gait: FGA Fitness: 6MST	14 h, 2 × 60 min p.w for 7 wk.
Eggenberger (2015) [27]	*N* = 89 IG1:*n* = 30, 77.3 ± 6.3,n.a. IG2:*n* = 29, 78.5 ± 5.1,n.a. IG3:*n* = 30, 80.8 ± 4.7,n.a.	comparison of effects of physical MCT training to a stepping-based Exergame on cognition	IG1:GDT-EXG (Stepmania) IG2:GDT- EXG (Memory) IG3:PHY (Walking)	**EF/SH**(vi-mo):TMT-B **WM:** Paired-Associates Learning Task; **ATT**: Age concentration test A and B; **PS** (vi-mo) TMT-A, DSST and WAIS-R; **GCF**: MoCA	n.a.	36 h / 3 × 60 min p.w. for 3 12 wk. / 24 wk.
Eggenberger (2016) [33]	*N* = 33; IG:*n* = 19, 72.8 ± 5.9, 7 m/12f CG:*n* = 14 77.8 ± 7.4,5 m/9f	effects of DT video game against conventional balance training on PFC activity during walking and on EF	IG: GDT- EXG (Stepmania) CG:ST (Balance)	**EF/SH** (vi-ma) TMT-B **ATT/ INH** (vi-ma) Stroop test **GCF**: MoCA	lower extremity function: SPPB	18 h / 3 × 30 min p.w. for 12 wk.
Falbo (2016) [15]	*N* = 36; IG: *n* = 20,71.5 ± 6.7, 2 m/18f CG: *n* = 16,73.7 ± 4.5, 2 m/14 f	benefits of a DT training specifically on EF compared to physical training with lower executive demands	IG: GDT CT: ST	**EF** (A-ve): verbal RNG Test **INH** (A-ve) Turning point index, adjacency, runs **WM** (A-ve): redundancy, coupon, repetition gap	walking ST and DT: speed, gait length and cadence recording with photocell system	24 h / 2 × 60 min p.w. for 12 wk.
Hars (2014) [34]	*N* = 134; IG:*n* = 66,75 ± 8, 2 m/64f CG: *n* = 68, 76 ± 6,3 m/65f	effects of a multi-task music based training on cognitive functioning and mood	IG: GDT CG: -	**GCF** (vi-ma): MMSE, CDT, FAB	n.a.	25 h/ 2 × 30 min p.w. for 25 wk.
Hiyamizu (2012) [35]	*N* = 43; IG: *n* = 21 71.2 ± 4.4,5 m/16f CG: *n* = 22, 72.9 ± 5.1,12 m/10f	effects of a DT balance training on postural control while performing a cognitive task	IG: GDT CG:ST (balance and strength)	**EF** (vi-ma): TMT (B-A) **INH**/**ATT** (vi-ve): Stroop task	Standing and walking, Chair Standing Test, TUGT, Functional Reach Test, COP displacements	24 h / 2 × 60 p.w. for 12 wk.
Kitazawa (2015) [36]	*N* = 60 G:*n* = 30, 76.8 ± 4.4, 17 m/13f, CG: *n* = 30, 75.5 ± 3.7, 10 m/20f	effect of a net-stepping exercise on cognitive performance and gait function	IG: GDT CG: -	**GCF:** Touch Panel-Type Demetia Assesment Scale (TDAS)	Mobility: TUG	8 h / 1 × 60 min p.w. for 8 wk.
MacLean (2014) [37]	*N* = 45; IG1:*n* = 15, 73.2 ± 5.36,4 m/11f IG2: *n* = 15, 69.1 ± 3.37,6 m/9f IG3: *n* = 15, 72.9 ± 6.49,7 m/8f	effects of musical training on the gait and cognition in healthy older people	IG1: GDT IG2: ST IG3: ST	**GCF**: MMSE; **EF/ SH/ PS:** TMT A/B; **WM** (ve) DS b/f; Immediate and delayed story recall	Gait (ST/DT conditions): velocity, stability	n.a.
Malliot (2012) [29]	*N* = 32, IG *n* = 16, 73.47 ± 4.10 CG *n* = 16, 73.47 ± 3.0; 5 m/27f	determine whether exergame training sport activity would show transfer to cognitive functions	IG:GDT- EXG CG: -	**EF/INH/ ATT** (vi-ve): Stroop Test, TMT A/B. Matrix Reasoning Test; **WM** (vi-ve): Spatial Span Test b/f, Directional Headings, Mental Rotation Letter Digit,; **PS**: cancellation test, number comparison test; PS(vi-ma): Reaction time test, Plate Tapping test	Heart rate (6 min walking); Chair Stand 8-ft-up and go; Arm curls, “back scratch”	12 h/ 1 × 60 min p.w. for 12 wk.
Nishiguchi (2015) [38]	*N* = 48 IG: *n* = 24, 73.0 ± 4.8, 13 m/11 fm CG: *n* = 24, 73.5 ± 5.6, 13 m/11f	investigate whether a physical and cognitive program can improve cognitive function and brain activation efficiency in older people	IG: GDT CG: -	**GCF**: MMSE **WM**: WMS-R **EF/ATT PS:** (vi): TMT B-A, Go-No-Go-Stimulus, Test off attentional control	10-m walking test, TUGT, 5-CtST	18 h/ 1 × 90 minp.w. for 12 wk.
Ordnung (2017) [30]	*N* = 30 IG: *n* = 15, 69 ± 79 6.34, n.a. CG: *n* = 15, 68 ± 4.67, n.a.	investigate the effect of a whole-body Exergame training intervention	IG: GDT- EXG CG:-	**ATT/PS**: Test of Attentional Performance; **WM** (vi-ma): n-back task 2; **INH**: Go/ No-Go-Stimulus	3 min. Step Test, upper body muscle endurance test, grip strength, hand motor skills; Motor RT: Ruler Drop Test, Balance (Balance Board)	12 h/ 2 × 60 min p.w. for 6 wk.
Schaettin (2016) [39]	*N* = 27; Mean 80 IG1: *n* = 13, 8 m/5f IG2: *n* = 14, 80, 7 m/7f	compare Exergame training with conventional balance training	IG1: SDT- EXG IG2: Balance	**EF** (vi-ma):computerized TAP (WM, SHI, divided ATT, INH (Go−/No-Go-task))	n.a.	15 h/ 3 × 30 min p.w. for 10 wk.
Schoene (2013) [31]	*N* = 37 IG: *n* = 15, 77.5 ± 4.5, n.a. CG; *n* = 17, 78.4 ± 4.5,n.a.	effects of a stepping exergae on stepping performance and associated fall risk	IG: SDT- EXG CG: -	**PS** (vi-mo): Choice Stepping Reaction time; **EF:** TMT; **DTC**:TUG-DT	Physiological Profile Assessment (Fall Risk); TUGT, 5STS, Alternate Step Test (AST	16 h/ 3 × 20 min p.w. for 16 wk.
Schoene (2015) [40]	*N* = 90 IG: *n* = 47, 82 ± 7, 66%f CG: *n* = 43, 81 ± 7,67%f	effectiveness of step-based exercise game on cognitive functions associated with falls	IG: SDT- EXG CG: education brochure	**EF**(vi-mo): Stroop Stepping Test (SST); **PS/ATT (**vi-mo): Letter-digit test, CRT + CSRT; TMT A, Attentional network test; **INH:** Victoria Stroop Task **DTC:**TUG-DT; **WM**: Digit span B;	n.a.	16 h // 3 × 20 min p.w. for 16 wk.
Wollesen (2017a) [41]	*N* = 95 IG1: *n* = 26,72.2 ± 4.6,10 m/16f, IG2: *n* = 30, 69.8 ± 5.7,2 m/28f CG1: *n* = 19, 72.9 ± 4.4, m/12f . CG2: *n* = 20, 72.7 ± 5.3, 3 m/17f	effects of a DT training in people with and without concern about falling on walking performance	IG1: GDT (FES-I < 20) IG2: GDT (FES-I > 20) CG1: - (FES-I < 20) CG2: - (FES-I > 20)	**DT/ATT** performance (vi-ve): Stroop Task	Walking performance: ST =30-s walking test DT: 30-s-walking test + (vi-ve) Stroop Task	12 h1 × 60 min p.w. for 12 wk.
Wollesen (2017b) [42]	*N* = 78 IG1: *n* = 29,70.7 ± 4.9, 7 m/22f IG2: *n* = 23, 71.7 ± 4.9,8 m/15f CG: *n* = 26, 73.7 ± 5.0,7 m/19f	effects of a DT balancetraining and a ST strength and resistance training on motor performance during DT walking	IG1: GDT IG2: PHY (Strength and resistance) CG: -	**ATT/DT performance** (au-ve): Stroop task while walking	Walking performance: ST and DT conditions	12 h/ 1 × 60 min p.w. for 12 wk.
You (2009) [43]	*N* = 13 IG: *n* = 8,70.5 ± 6.8,2 m/11f CG: *n* = 5, 68.0 ± 3.3, 1 m/4f	determine long-term practice effects of CGI on cognition and gait performance in older people with a history of falls	IG: SDT CG: ST (walking)	Memory recall (ve): memorise and recall words	walking performance under DT conditions: velocity; stability; COP displacements	15 h/ 5 × 30 min p.w. for 6 wk.
**Controlled clinical trials and other**						
Ansai (2017) [25]	*N* = 80; IG: *n* = 41,68.5 ± 8.4, n.a. CG:*n* = 39, 68.5 ± 6.3; n.a.	effects of the addition of a dual task to MCT on cognition	IG: TDT (MCT + CT), CG: MCT	**GCF:** MMSE, MoCA; **VS:** CDT; EF (Ve-mo): **DTC:** TUGT-DT memorising number and dialling while walking	TUGT: mobility	60 h/ 3 × 50 min p.w. for 12 wk.
Bisson (2007) [44]	*N* = 24; IG1: *n* = 12; 74. ± 3.6,7 m/5f IG2: *n* = 12, 74 ± 4.92,2 m/9f	determine the effect of VR and BF training on balance and reaction time in older people	IG1: GDT- EXG (VR) IG2: GDT- EXG (BF)	**PS** (au-ve): verbal reaction to auditory cue	COP displacement; CB&M Scale	10 h/ 2 × 30 min p.w. for 10 wk.
Chuang (2015) [45]	*N* = 26, IG1:*n* = 7, 69.43 ± 3.82, IG2:*n* = 11, 67.01 ± 1.67, CG:*n* = 8, 68.25 ± 3.96; 26f/0 m	examine whether DDR training would exert similar effect on interference control as that brisk walking in elderly individual	IG1: SDT- EXG (DDR) IG2: ST (walking) CG: -	**EF** (Vi-mo): reaction to visual stimulus on screen in reaction time/ms; EEG recording **INH**: Flanker Test	n.a.	18 h/ 3 × 30 min p.w. for 12 wk.
Heiden (2010) [46]	*N* = 16, Mean Age 77 IG *n* = 9, 5 f/ 4 m CG = 7, 6 f/ 1 m	Effects of a games-based balance training program on general fitness and attentional demands in postural control	IG: GDT CT: -	**PS** (A-Ve): reaction on auditory simulus	CB&M Scale, 6 min walking, COP displacements (RMS)	8 h/ 2 × 30 min p.w. for 8 wk.
Kayama (2014) [28]	*n* = 41; > 65 IG: *n* = 26, n.a. CG: *n* = 15, n.a.	whether or not a DT Thai Chi training program would effectively improve cognitive functions	IG: ST+ EXG (GDT Thai Chi) CG: Standardised Training	**EF/PS**: TMT A/B **VF** (ve): Verbal Fluency Task	n.a.	60 h/ 1 × 80 min p.w. for 12 wk.
Morita (2018) [47]	*N* = 19 IG: *n* = 8, 75.0 ± 1.5, 8 m, CG: *n* = 11, 71.9 ± 4.0, 2 m/9f	effect of 2-year cognitive–motor dual-task (DT) training on cognitive functions and motor ability	IG: GDT CG: -	**GCF**: Modified Mini-Mental State(3MS) **PS/ATT** (vi): TMT A/B	Quadriceps isometric muscle strength motor ability: TUGT, maximal step length (MSL)	104 h/ 1 × 60 min p.w. for 104 wk
Theill (2013) [48]	*N* = 63 IG1: *n* = 21, 72.39 ± 4.19, IG2: *n* = 16, 73.33 ± 6.08, CG: *n* = 26, 70.90 ± 4.77,	effects of simultaneously performed WM and PHY training on cognitive and motor-cognitive dual task performance	IG1: GDT IG2: ST (cognitive training) CG: -	paired associates learning; **ATT** (vi-ma): continuous performance task **EF**:sequential learning **PS**(vi-ma): Digit-letter task **WM** (ve): n-nack task	Walking ST/DT conditions	13 h/ 2 × 40 min p.w. for 10 wk.

**Legend: Participants**: *IG* intervention group, *CG* control group, *f* female, *m* male, *MMSE* Mini Mental Status Examination, *MoCA* Montreal Cognitive Assesment; **Intervention**: *CL* Cognitive load, *KAG* kinect adventures games, *CPT* conventional physical therapy, *MT* Motor task, *MCT* Multicomponent Training, *DT* Dual Tasking, *ST* Single Tasking, *GDT* General Dual-Tasking, *SDT* Specific Dual-Tasking, *EXG* Exergaming, *CT* Computerized training, *VR* Virtual Reality, *BF* Biofeedback, *PHY* Physical Training; **Cognitive assessment:***GCF* Global cognitive function, *PS* Processing Speed, *Sh* Shifting, *EF* Executive Function, *INH* Inhibition, *WM* Working Memory, *ATT* Attention, VS Visuospatial, *DTC* Dual-Task Costs, **Stimulus-response**: *A-Ve* Auditory Verbal, *A-ma* auditory manual, *Vi-Ve* Visual-verbal, *Vi-mo* Visual-motor, *Ve-mo* Verbal-motor, A**ssessments**: *TUGT-DT* Timed up and go test with dual tasking, motor task, *TUGT* Timed up and go test, *TMT A/B* Trail Marking test A/B, *DS B/F* Digit Symbol backwards/forwards, *CSRT* Choice Stepping Reaction Time, **Training duration**: *p.w.* per week., *wk* week

### Quality assessment and bias

Based on the quality assessment, 17 articles were considered to be of high quality, five of fair quality and only three articles of low quality. Eighteen of the included articles were RCTs, of which 15 were of high and three of fair quality. Two of the seven controlled trials were rated of high quality, two of fair quality and three of low quality. In general, most studies reported the in/exclusion criteria (92%), comparable baseline characteristic for the control and intervention groups on the most important prognostic indicators (80%), and group comparisons (80%). All studies provided a measurement of variability for at least one key outcome. Only three studies (12%) blinded their participants. Four studies (16%) reported blinding of the therapists who administered the exercise intervention, and only three studies trials (12%) reported blinding of all assessors for at least one key outcome (cf. Table 2).

**Table 2:** Quality assessment of included studies according to PEDro scale

Study	Quality criteria											Quality Score
	1	2	3	4	5	6	7	8	9	10	11	
Ansai [25]	x	–	–	–	–	–	–	–	x	x	x	**4**
Azadian [26]	–	x	–	x	–	–	–	x	x	x	x	**6**
Bacha [32]	x	x	x	x	–	–	–	x	x	–	x	**7**
Bisson [44]	x	–	–	–	–	x	–	–	–	x	x	**4**
Chuang [45]	x	–	–	x	–	–	–	x	x	x	x	**6**
Eggenberger 2015 [27]	x	x	–	x	x	–	–	x	x	(x)	x	**7**
Eggenberger 2016 [33]	x	x	x	x	x	–	–	–	x	x	x	**8**
Falbo [15]	x	x	–	–	–	–	–	–	x	–	x	**4**
Hars [34]	x	x	–	x	–	–	x	–	x	x	x	**7**
Heiden [46]	x	–	–	–	–	–	–	(x)	–	x	x	**3**
Hiyamizu [35]	x	x	(x)	(x)	–	x	–	–	–	x	x	**5**
Kayama [28]	x	–	–	–	–	–	–	x	–	(x)	x	**3**
Kitazawa [36]	x	(x)	–	x	–	–	–	x	x	x	x	**6**
MacLean [37]	x	x	–	x	–	–	–	–	x	x	x	**6**
Maillot [29]	x	x	–	x	–	–	–	x	x	–	x	**6**
Morita [47]	x	–	–	x	–	–	x	x	x	–	x	**6**
Nishiguchi [38]	x	x	(x)	x	–	x	–	x	x	–	x	**7**
Ordnung [30]	x	x	–	x	–	–	–	x	x	x	x	**7**
Schaettin 2016 [39]	x	x	–	x	x	–	–	x	x	x	x	**8**
Schoene 2013 [31]	x	x	–	x	–	–	_	x	x	x	x	**7**
Schoene 2015 [40]	x	x	(x)	x	–	x	x	(x)	x	x	x	**8**
Theill [48]	–	–	–	x	–	–	–	–	–	x	x	**3**
Wollesen 2017a [41]	x	x	–	x	–	–	–	x	x	x	x	**7**
Wollesen 2017b [42]	x	x	–	x	–	–	–	x	x	x	x	**7**
You [43]	x	(x)	–	x	–	–	–	x	x	(x)	x	**5**

**Legend: 1** - eligibility criteria were specified. 2 - participants were randomly allocated to groups. 3 - allocation was concealed. “4 - the groups were similar at baseline regarding the most important prognostic indicators”. 5 - there was blinding of all participants. 6 - there was blinding of all therapists who administered the therapy. 7 - there was blinding of all assessors who measured at least one key outcome. “8 - measures of at least one key outcome were obtained from more than 85% of the participants initially allocated to groups”. “9 - all participants for whom outcome measures were available received the treatment or control condition as allocated or, where this was not the case, data for at least one key outcome was analyzed by “intention to treat””. “10 - the results of between-group statistical comparisons are reported for at least one key outcome”. “11 - the study provides both point measures and measures of variability for at least one key outcome”. x – “yes” score.. “-” – “no” score. (x)- undertaken with general remarks

### Intervention characteristics

The intervention characteristics, including the type of intervention, a description of the motor and cognitive components, frequency and dose are outlined in Table 3.

The cognitive load, which was combined with the motor exercise, showed different levels of cognitive demand. Exergaming situations differed between commercially available computer games to laboratory-based training forms. In many cases the digital game required players to interact with objects on a screen in the virtual environment, which could be moved or controlled by bodily movements. A cognitive load was also provided, when the intervention required the participant to work with a visual feedback or biofeedback as part of the technology-based game.

The cognitive-motor components differed between the evaluated studies. Fourteen trials used a traditional dual-task intervention and eleven trials used modern technology or a combination of traditional and technology-based interventions. The included studies show a range of interventions, which differed in both motor exercise and cognitive load of the additional cognitive task. Motor interventions varied from specific walking or stepping tasks to general balance and strength training (cf. Table 3). Most trials included different additional cognitive tasks to the motor components (e.g. working memory tasks, verbal fluency tasks, visual search tasks) and can be named as GDT intervention. Five out of 25 studies used a SDT training (e.g. body shifting to control a virtual paddle) to examine the influence of a cognitive-motor training on cognition [31, 39, 40, 46].

**Table 3:** Data extraction of included studies

Author	Training intervention	Motor-component	Cognitive component	Cognitive measurement	Progression	Control group(s)	Results
Ansai [25]	GDT	Warm up, muscle strengthening, balance, coordination, flexibility	Working memory, inhibition	MMSE (main scores and subscales) MoCA (main scores and subscales), TUG-DT	In the complexity of the cognitive task	Physical exercises without DT	No differences between the groups regarding the cognitive outcomes; the MMSE and the visuo-spatial test of the MoCA increased; DTC decreased
Azaidian [26]	GDT	Standing and shifting center of gravity Walking exercises to the front, backwards and sides	Working memory tasks Verbal fluency tasks Visual search tasks	Reaction time while (1) sitting, (2) standing, (3) walking, (4) selective (respond to direction of task) Stop Signal Task to measure inhibitory control Working memory with Wechsler Adult Intelligence scale and Digital Symbol substitution test	Session 1–6 only motor training Session 7–12 motor training with simple cognitive tasks Session 13–24 Task complexity increased	CG 1: computer- based EF training CG 2: no intervention	GDT training only improved the Wechsler forward in comparison to control groups EF training improved SST correct answers and wrong answers; stride asymmetry while DT walking
Bacha [32]	GDT-EX Xbox Kinect adventure game	Fast multidirectional movements (steps, squats, jumps, coordinated movements of upper and lower limbs; trunk movements in three planes	Reaction time; visuospatial attention, shifting of attention, decision making, immediate planning and execution	MoCA	Not reported	conventional physiotherapy including balance, endurance and muscle strength, motor coordination; stretching	Both groups increased within all performance measurements; the control group increased walking capacity
Bisson [44]	Specific Virtual reality DT training	Jiggle a virtual ball while standing	Reaction time; visuospatial attention, immediate planning and execution	Reaction Time test	Not reported	Biofeedback training with shifting the center of mass	No significant group differences; both groups improved in the cognitive task
Chuang [45]	GDT-EX - video dance	Stepping forward, backwards and sidewards according to the music and presented steps on a screen (following an arrow)	Reaction time, Attention and visuo-spatial orientation	Flanker task	Not reported	CG 1: brisk walking CG 2: inactive	Reaction times decreased in the intervention group as well as in the brisk walking group
Eggenberger 2015 [27]	GDT-EX - video dance	Stepping forward, backwards and sidewards according to the music and presented steps on a screen (following an arrow)	Attention; reaction time and visuo-spatial orientation	EF: Trail Making B Long-term visual memory, Long-term verbal memory (story recall) Wechsler Memory scale revised	Progression adapted to participants abilities	CG1: treadmill walking memory CG2: walking	Both DT training groups improved the TMT-B; over a longer period of time the Dance group still improved whereas the memory group declined; same results for the executive control tasks; GDT-EX improved Working memory, attentional control; Go/no-go and set shifting
Eggenberger 2016 [33]	GDT-EX - video dance	Stepping forward, backwards and sidewards according to the music and presented steps on a screen (following an arrow)	Attention; reaction time and visuo-spatial orientation	EF: Trail Making B Stroop task Working memory task MoCa Processing speed	Progression adapted to participants abilities	Balance training on different surfaces	The intervention group improved the Trail making B, MoCA and the Stroop task
Falbo [15]	GDT	Physical- cognitive DT training; walking at different speeds; coordination training, balance performance; strengthening, stretching with music together with different cognitive tasks	Inhibition, working memory and set-shifting	Random number generation task to address EF; dual task cost while walking	Rising difficulties (not further described)	Same exercise program under single task condition	The GDT group improved cognitive function
Hars [34]	GDT with music	Walking and handling of objects; reaction to the rhythm of the music	Reaction time	MMSE; Frontal assessment battery (FAB)	Progression mentioned but not further described	No intervention	Intervention group increased MMSE
Heiden [46]	SDT balance	Body shifting to control virtual paddle	Reaction time; visuospatial attention, immediate planning and execution	Reaction time	Chair based exercise with muscle strengthening	Reaction time decreased in the intervention group	
Hiyamizu [35]	GDT balance	Strength training, balance and walking training using different undergrounds in combination with verbal fluency, arithmetic and visual search task	Working memory Visuospatial tasks	Trail making A and B Stroop task	Not reported	Same program but ST	Only Stroop task performance improved in the GDT group
Kayama [28]	GDT and SDT (exercises with specific Dual task Tai Chi)	Aerobic training, progressive muscle strengthening, flexibility and balance; rhythmic stepping exercise with cognition; 5 min Dual task Tai Chi at the end	Unclear; Dual task Tai Chi includes visuospatial tasks	Verbal fluency test; Trail making B	Only reported for strengthening	Same training than intervention group without Dual task Tai Chi	the intervention group improved the Delta TMT
Kitazewa [36]	SDT net step exercises	Steps within a net in a predefined way; every session learning a new combination; avoid to step on the net; than performing a line with steps in the net while singing a children song	Working memory task	Touch panel type dementia scale; Touch M system addresses visuospatial function; the TDAS is a modification of the Alzheimer’s Desease Asssessment Scale	Increasing of steps and difficulty of the combination	No intervention	Thouch M score increased more in the intervention group; Naming fingers as part of the TDAS improved in the intervention group
MacLean [37]	SDT	Walking with adjusting to the speed of music; ST walking, music walking; DT walking with music and counting backwards	Working memory	MMSE; TMT A-B; Wechsler memory scale revised Digit span forward and backward; Story recall DT walking	Not reported	CG1: walking to music without adjusting CG2: walking without music	MT training improved DT walking
Maillot [29]	GDT-EX (Nintendo Wii)	Body shifting and arm movements in front of the screen or on the Wii balance board	Visuo spatial tasks Processing speed tasks	TMT A-B, Stroop test Letter set tests Matrix reasoning test Digit symbol substation test Spatial span test Directional heading test Mental rotation test Cancellation test Number comparison test Reaction time test Plate tapping test	Not reported	Non active	the intervention group improved in all cognitive tasks except of the visuo spatial tasks
Morita [47]	GDT	Mental gymnastics with complicated finger movements; resistance training with DT, aerobic exercises with changing movement directions and DT; flexibility exercises	Working memory; reaction time	Modified minimental State (3MS) TMT with a touch panel	Not reported	Not active	Intervention group maintained cognitive status whereas control group decreased
Nischiguschi [38]	GDT	Stretching, muscle strength, DT categories (working memory, reaction time, visuospatial tasks)	Working memory, reaction time Visuospatial tasks	MMSE; Wechsler memory scale revised TMT A-B; N-Back	Reported for strength training but no further details	No intervention	Intervention group better results in WMS-R and TMT
Ordnung [30]	GDT- EX X box™360 Kinect™	Whole-body movements to move an avatar on screen	Attention, visuospatial function, reaction time Shifting and decision making	Attention while being seated with Test of Attentional Performance; Simple reaction time/Alertness while being seated in front of a computer: response (finger pressing) to a visual stimuli on screen; Working memory (seated) with the n-back task)	Not reported	No intervention	No significant improvement in tested cognitive functions, but improvements in fine motor skills of the left hand
Schaettin [39]	GDT-EX	lower extremity movements, stepping according to force platform	Attention; reaction time and visuo-spatial orientation	Attention, inhibition, working memory (Test for attentional performance), Cognitive Function (MMSE)	Not reported (warm up 5 min; training 20 min, cool down 5 min)	CG: traditional balance training, static and dynamic exercises, open eyes and closed eyes	Four EF’s increased in the EXG group and one (shifting) in the CG
Schoene 2013 [31]	SDT-EX Dance training	Standing, stepping, weight shifting	Attention; reaction time and visuo-spatial orientation	Processing speed (Choice stepping reaction time; TMT A), shifting (TMT B), Dual-task costs (TUG-cog)	Frist session supervised by an instructor, follow-up sessions individualized sessions in homes	CG: no intervention	Improvement in step reaction and movement times
Schoene 2015 [40]	SDT-EX Dance training	Standing, stepping, weight shifting	Attention; reaction time and visuo-spatial orientation	Inhibition (Stoop Stepping Test); Working Memory (letter-digit test, digit span backwards), Processing speed/ Attention (Test for attentional network test, TMTA, CRT+ CSRT; shifting (TMT B); Dual task costs (TUG-cog)	Instruction at the beginning of the trial; conduction unsupervised in individuals’ homes	CG: educational brochure in falls prevention	IG improvement in processing speed and mental rotation, set- shifting increased with a higher dose of game playing; individuals with poorer baseline function in IG showed greater improvement
Theill [48]	GDT	Cardiovascular treadmill training; walking	Verbal Working memory	(selective) Attention, working memory, paired associates learning, processing speed, Dual task costs	Not reported	IG2: working memory training (single) CG: no intervention	Improvement in executive control, no improvement in selective attention, more improvement in IG in paired associates learning
Wollesen 2017a [41]	GDT	Standing, balancing, muscle training	Working memory, reaction time Visuospatial tasks; task prioritization, task shifting	Dual tasks costs (walking under DT and ST conditions), Inhibition (seated Stroop Test/ walking while undertaking Stroop Test)	Two phase intervention: Phase 1 (wk. 1–6); training of daily actions with likelihood of fall risks; Phase 2 (wk. 7–12) Task priorization	IG2 single task strength and resistance CG: no intervention	No significant improvement in IG1 in cognitive functions
Wollesen 2017b [42]	GDT	Walking, standing, balancing,	Working memory, reaction time Visuospatial tasks; task prioritization, task shifting	Dual-task costs (walking under ST and DT conditions), Inhibition (verbal Stroop task)	Two phase intervention: Phase 1 (wk. 1–6); training of daily actions with likelihood of fall risks; Phase 2 (wk. 7–12) Task priorization and transfer into daily life	IG2: ST conditions CG: no intervention	Reduced number of errors in IG in Stroop test
You [43]	SDT	Walking, standing	Memorizing	Working memory (memory recall %)	Not reported	CG: no intervention	Memory performance improved under DT conditions

### Results on cognition

The intervention results, including effects on global cognition, global executive function, mental flexibility, inhibitory control, working memory, visuospatial planning, verbal fluency, attentional control, processing speed as well as dual-task costs are outlined in Tables 3 and 4.

**Table 4:** Data extraction - Results on different cognitive dimensions

Author Year	GDT / SDT / EXG	Global cognitive function	set-shifting	inhibitory control	working memory	visuospatial planning	verbal fluency	attentional control	processing speed	dual-task costs
Ansai [25]	GDT	n.s	n.a.	n.a.	n.a	n.s.	n.a	n.s	n.a	↓
Azadian [26]	GDT	n.a.	n.a.	IG2:↑; IG1: n.s.	IG1: ↑	n.a.	n.a.	n.a.	n.s.	n.a.
Bacha [32]	GDT- EXG	IG + CG↑	n.a.	n.a.	n.a.	n.a	n.a..	n.a.	n.a..	n.a.
Bisson [44]	GDT- EXG	n.a.	n.a.	n.a.	n.a	n.a.	n.a	n.a.	IG1 + 2 ↑	n.a.
Chuang [45]	SDT- EXG	n.a.	n.a.	n.s.	n.a	n.a.	n.a	n.a.	RT IG: ↑ RT CG: ↓	n.a
Eggenberger 2015 [27]	GDT -EXG	n.a.	IG 1+ IG 2: ↑	n.a.	IG 1 + IG2: ↑	n.a.	n.a	IG 1 + IG2: ↑	↑	n.a
Eggenberger 2016 [33]	GDT- EXG	n.s	↑	n.a.	n.a	n.a.	n.a	n.a.	↑	n.a
Falbo [15]	GDT	n.a.	IG:↑ CG1: ↓	n.s.	n.s.	n.a.	n.a	n.a.	n.a	n.a
Hars [34]	GDT	↑	n.a.	n.a.	n.a	n.a.	n.a.	n.a.	n.a	n.a.
Heiden [46]	GDT	n.a.	n.a	n.a.	n.a	n.a.	n.a	n.a.	IG: ↑	n.a.
Hiyamizu [35]	GDT	n.a.	n.s.	IG: ↑	n.a.	n.a.	n.a.	n.a.	n.s.	n.a.
Kayama [28]	SDT + EXG	n.a.	n.a.	n.a.	n.a.	n.a.	n.s.	n.a.	n.s.	n.a.
Kitazawa [36]	GDT	IG: ↑	n.a.	n.a.	n.a.	IG: ↑	n.a.	n.a.	n.a.	n.a.
MacLean [37]	GDT	n.a.	n.s.	n.a.	n.s.	n.a.	n.a.	n.a.	n.s.	n.a.
Malliot [29]	GDT-EXG	n.a.	IG: ↑	IG: ↑	IG: ↑	n.s.	n.a.	n.a.	IG: ↑	n.a.
Morita [47]	GDT	n.s.	n.s..	n.a.	n.a.	n.a.	n.s.	n.s.	n.a.	n.a
Nishiguchi [38]	GDT	n.s.	IG: ↑	n.a.	IG: ↑	n.a.	n.a.	IG: ↑	IG: ↑	n.a.
Ordnung [30]	GDT- EXG	n.a.	n.a.	n.s.	n.s.	n.a.	n.a.	n.a.	n.s.	n.a.
Schaettin 2016 [39]	SDT- EXG	n.a.	IG: ↑	IG: ↑	IG: ↑	n.a.	n.a.	IG: ↑	n.a.	n.a.
Schoene 2013 [31]	SDT- EXG	n.a.	n.a.	IG: ↑	n.a.	n.a.	n.a.	n.a.	IG: ↑	n.a.
Schoene 2015 [40]	SDT-EXG	n.a.	IG: ↑	n.s.	IG: ↑	n.a.	n.a.	↑	IG: ↑	n.a.
Theill [48]	GDT	n.a	n.a.	n.a.	IG 1: ↑	n.a.	n.a.	n.s.	n.a.	n.a.
Wollesen 2017a [41]	GDT	n.a.	n.a.	n.s.	n.a.	n.a.	n.a.	n.s.	n.a.	↓
Wollesen 2017b [42]	GDT	n.a.	n.a.	IG: ↑	n.a.	n.a.	n.a.	↑	n.a.	↓
You [43]	SDT	n.a.	n.a.	n.a.	IG: ↑	n.a.	n.a.	n.a.	n.a.	n.a.

*IG* Intervention Group, *CG* Control Group, *GDT* General dual-tasking, *SDT* Specific dual-tasking, *EXG* Exergame, ↑- performance increase, ↓- performance decrease, *n.s.* non significant, *n.a.* non available, *RT* Reaction time, *TMT* Trail Marking Test, Δ – delta score

#### Effects on global cognitive function

Seven studies examined the effects of training intervention on global cognitive function [25, 32–34, 36, 38, 47]. All training interventions were conducted as a GDT training and two used the exergaming approach [32, 33].

Five out of seven studies showed positive improvements of global cognitive function tests such as MMSE, MoCA or comparable instruments [32, 34, 36, 38, 47] (cf. Table 3). All but one of these studies used a GDT training method. Although all studies used a walking or stepping motor component, cognitive tasks were heterogeneous. While Hars and colleagues [34] required their participants to adapt their pace to the rhythm of the music, Kitazawa and colleagues [36] used a targeted stepping exercise while participants needed to recite a children’s song. Similarly, Nishiguchi and colleagues [38] delivered a walking exercise while conducting a verbal fluency task. Bacha and colleagues [32] showed positive effects on global cognitive function due to an exergame intervention, using the Xbox Kinect system, which required whole body movements for conduction of the virtual game on screen. The two studies that did not show a positive impact on global cognitive function used a GDT. The study, by Ansai and colleagues, with a total duration of 60 h (three times per week over 12 weeks), used a combination of a general balance, strength and walking training with a cognitive arithmetic task [25]. Eggenberger and colleagues [33] provided the Dance Dance Revolution GDT exergame without significant changes in general cognitive function with a duration of 18 h (three times per week 30 min for 12 weeks).

**Fig. 2:**
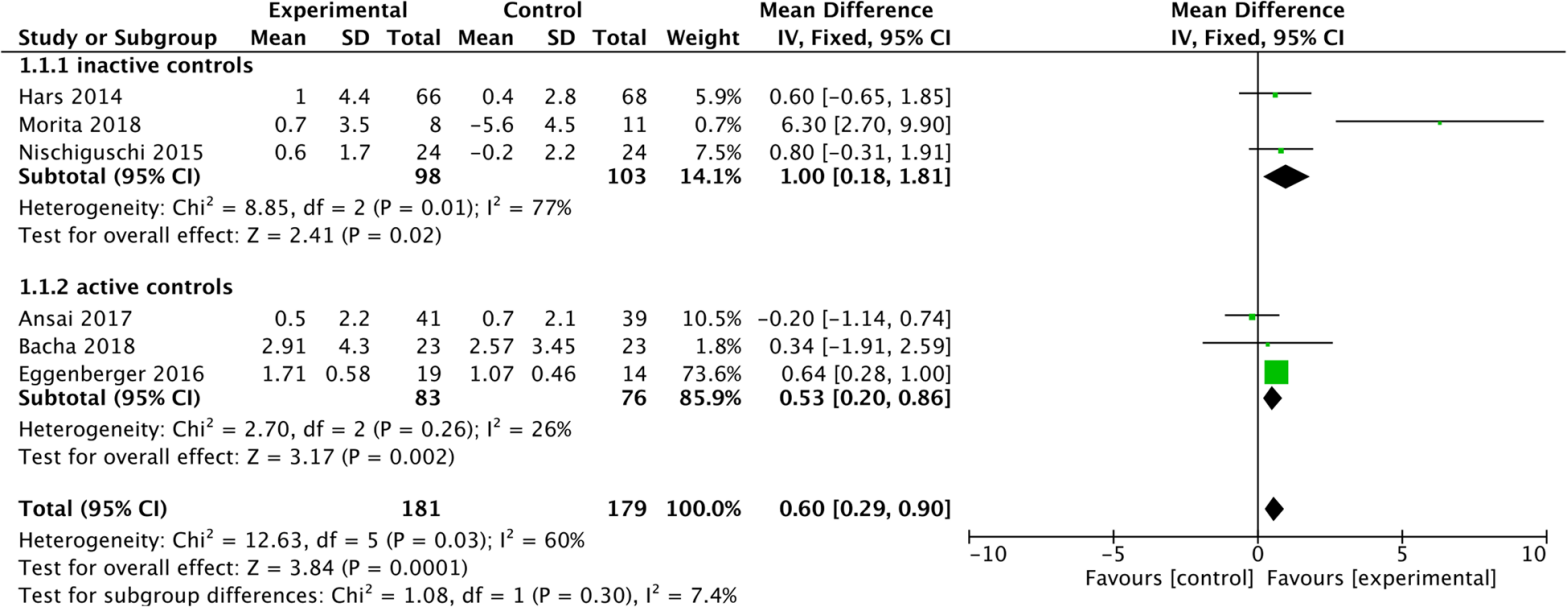
Training effects on global cognitive function

Six studies could be integrated into the meta-analysis (Fig. 2). Three of the studies had an inactive control group and three had an active control group (cf. Table 3).

Overall, the interventions showed a significant mean difference of 0.60 [95% CI 0.29–0.90] on global cognitive functioning. This effect was observed in comparison to active (mean difference 0.53) and inactive control groups (mean difference 1.0) with no significant difference for the control group conditions (cf. Fig. 2). However, these results were heterogeneous (I^2^ = 60%) due to the effects of the studies by Morita and colleagues [47] and Eggenberger and colleagues [33]. A sensitivity analysis excluding both studies revealed the effect for the comparison of the DT training intervention to an inactive control or active control group did not remain significant.

#### Effects on inhibitory control

Eleven studies evaluated training effects on inhibitory control, with six of these studies showing improvements [31, 33, 35, 41, 42]. Hiyamizu and colleagues [35] used a walking exercise combined with an alphabetical task while testing verbal fluency. Eggenberger and colleagues [33] and Schoene and colleagues [31, 40] used a dancing exergame for theirs trials, which required the participant to take a step in different directions following instructions on the screen, while controlling speed and inhibiting a step presented in a different colour. Wollesen and colleagues [41, 42] conducted a progressive task-managing training (e.g. task prioritisation and task switching) in combination with balance and walking exercises. These trials used SDT and GDT interventions and had a duration between twelve and 36 h. The remaining five studies showed no significant change in inhibitory control [15, 26, 30, 39, 45]. These trials used SDT and GDT interventions and had a duration between twelve and 24 h. However, the studies differed with regard to the activities of the control groups (cf. Table 3). A meta-analysis was conducted on seven of these studies.

**Fig. 3:**
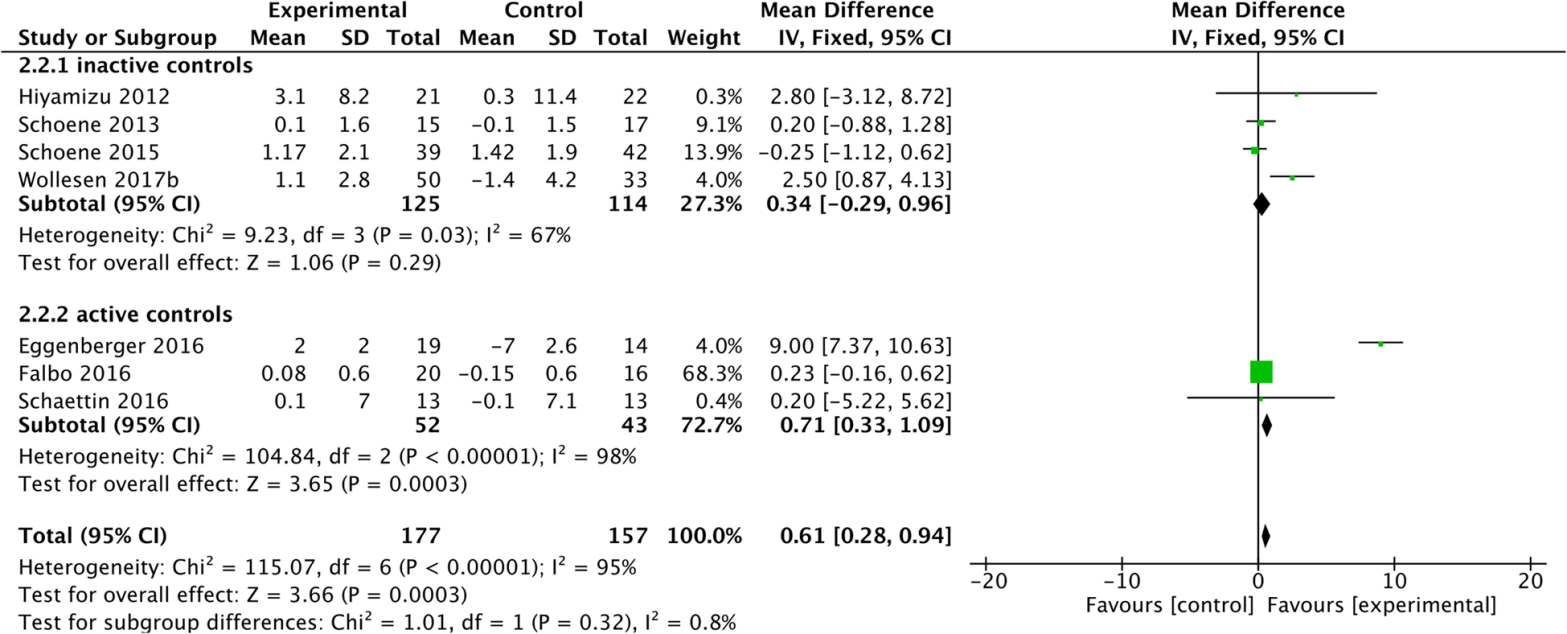
Training effects on inhibitory control

As shown in Fig. 3, studies with an active control group showed benefits on inhibitory control with a significant mean difference of 0.71 [95% CI 0.33–1.09], whereas the studies with inactive controls did not. However, heterogeneity was high with I^2^ = 95%. This large heterogeneity could have been a result of the data by Eggenberger and colleagues [33]. After running a sensitivity analysis without the study by Eggenberger and colleagues [33] the heterogeneity reduced to I^2^ = 46% and the effect between the training and the control groups did not remain significant.

#### Effects on working memory

Seven out of nine interventions reported improved working memory [26, 27, 30, 38, 40, 43, 48]. Three of these interventions were GDT exergames [27, 30, 40] three were GDT programs [26, 38, 43, 48] and You and colleagues [43] provided a DT walking training with additional working memory tasks. The interventions conducted by Falbo and colleagues [15] and MacLean and colleagues [37] did not increase the examined working memory task.

**Fig. 4:**
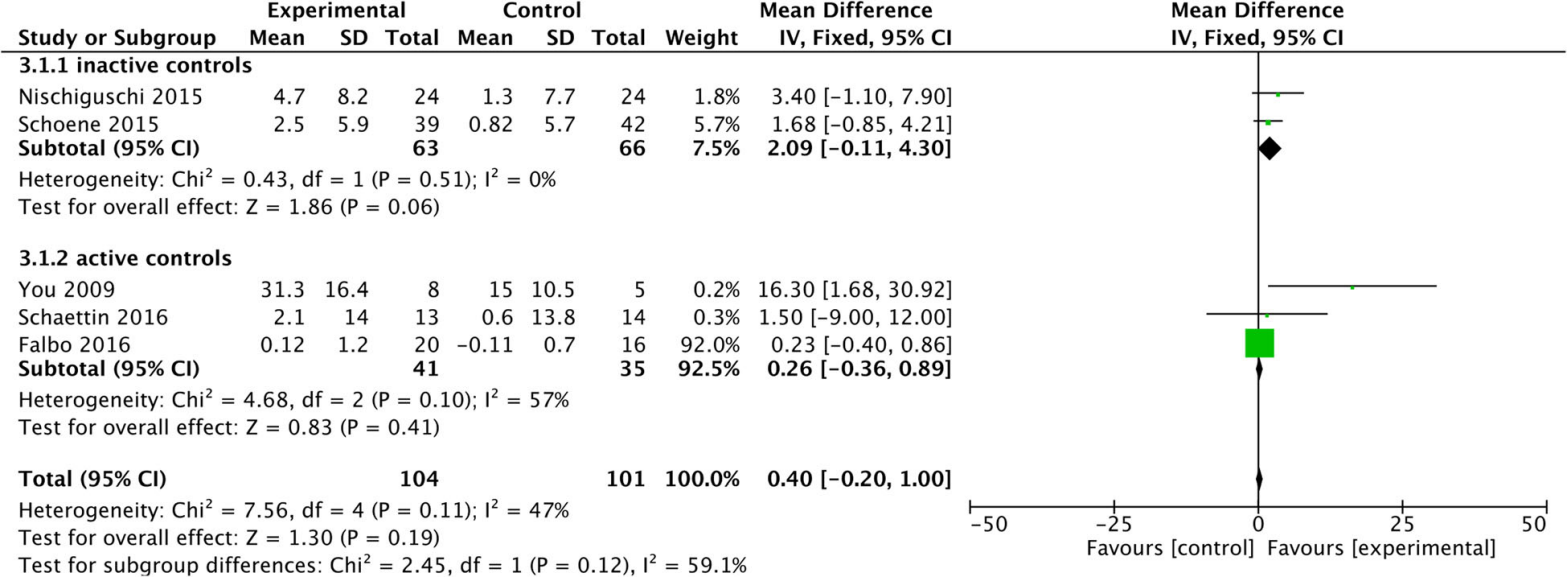
Training effects on working memory

The results of the meta-analysis for working memory showed a significant mean difference of 2.09 [95% CI -0.1-4.30] of the two studies who had an inactive control group. The comparison of a DT intervention with physical active controls did not show advantages for the DT training on improving working memory. In addition, there was no overall effect on working memory for the integrated five studies in the meta-analysis (cf. Fig. 4). Heterogeneity was moderate (I2 = 47%), mainly caused by the studies with an active control group (I2 = 57%).

#### Effects on cognitive flexibility (i.e. set-shifting)

**Fig. 5:**
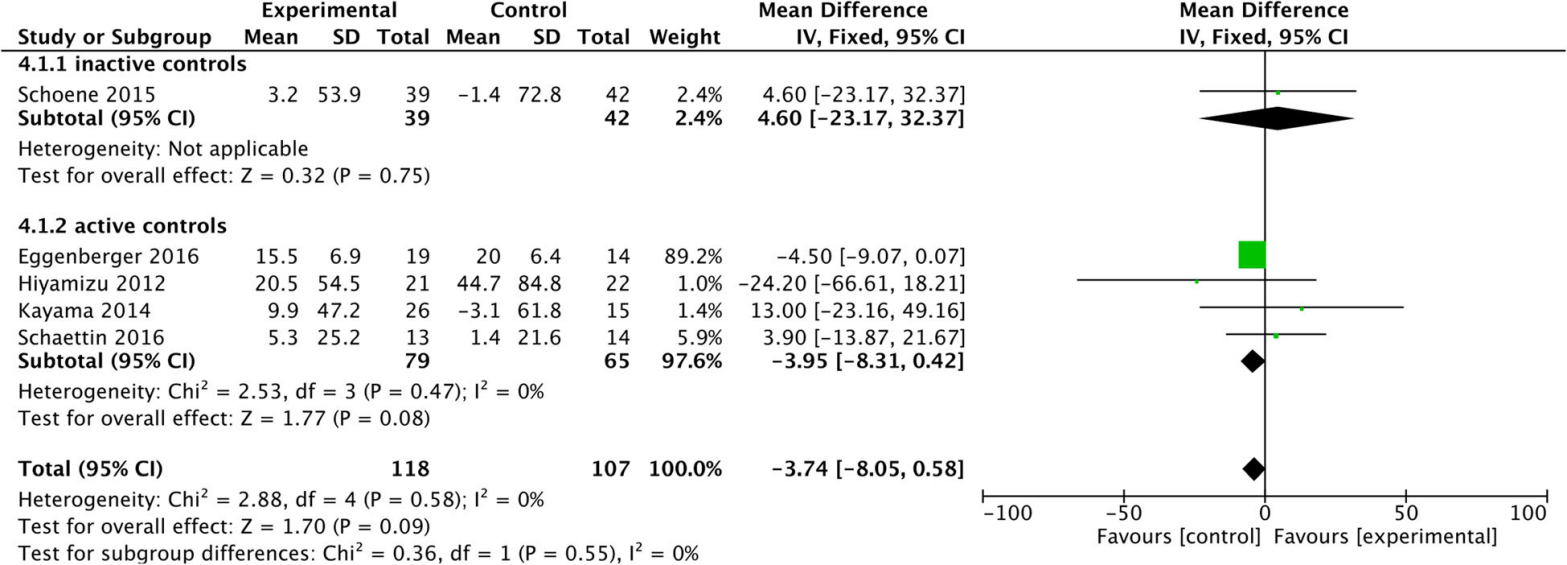
Training effects on cognitive flexibility

Seven out of ten interventions showed improved set-shifting after a GDT or a GDT exergame intervention, using the TMT A/B, TMT B-A or the verbal random number generation test (RNG) [15, 27, 29, 33, 35, 38, 40]. The training duration of these interventions was between twelve and 36 h. The three interventions by Kayama and colleagues [28] (a GDT in combination with SDT Tai Chi), MacLean and colleagues [37] (SDT with walking to music) and Schaettin and colleagues [39] (GDT exergame with stepping on a force platform) did not increase set-shifting abilities. The meta-analysis of five studies that could be added into the analysis did not show any effects of the DT interventions (cf. Fig. 5).

#### Effects on visuospatial planning

Four studies intended to improve visuospatial planning by using GDT [25, 29, 36, 40]. Only the programs run by Kitazawa and colleagues [36] and Schoene and colleagues [40] improved visuospatial planning.

#### Effects on attentional control

Attentional control was examined in nine of the training studies. Seven of these led to significant improvements of attentional control. Four of these studies used a GDT intervention [35, 38, 41, 42, 47]. Two studies conducted a SDT exergame intervention [39, 40] and one used a GDT exergame [27].

The GDT programs by Ansai and colleagues [25] and Theill and colleagues [48] failed to show effects on attentional control. Both studies conducted a GDT program, which required the participants to engage in walking as well as resistance training interventions combined with a numerical task or a cognitive load.

#### Effects on processing speed

Processing speed was the most examined variable within the cognitive tasks of the included studies. A total number of 15 studies focused on this outcome. All exergaming interventions (*n* = 8) except of the one by Kayama and colleagues [28] showed improvement of processing speed [27, 29–31, 33, 40, 44, 45]. Moreover, the two GDT programs by Heiden & Lajoie [46], and Nishiguschi and colleagues [38] improved processing speed.

In contrast the GDT programs by Azadian and colleagues [26], Hiyamizu and colleagues [35], MacLean and colleagues [37] and Morita and colleagues [47] did not affect processing speed. All trials used a GDT walking intervention, which was combined with different cognitive tasks, e.g. counting backwards or naming out of memory. MacLean and colleagues [37] and Hars and colleagues [34] both engaged their participants in a pace adaption task, which required participants to adjust the speed of their pace to the rhythm of the music played along the walking exercise.

#### Effects on DTC

The studies by Ansai and colleagues [25]; MacLean and colleagues [37] and Wollesen and colleagues [42] also addressed cognitive-motor interference by analysing DTCs. All programs were able to reduce cognitive-motor interference, leading to improved performance on the cognitive or motor task.

#### Influence of training methods

Overall, 24 interventions were able to improve at least one cognitive outcome. The majority of the studies increased the cognitive outcome that was specific to the training intervention (cf. Table 3). Three studies were not able to show effects on their cognitive test battery [25, 30, 47]. The studies conducted by Ansai and colleagues [25] and Morita and colleagues [47] integrated a cognitive test that was not correlated to the cognitive dimensions that were addressed in the training program.

Moreover, seven studies showed improvement of cognitive abilities that were specifically addressed as a part of the training intervention. Three of them were exergaming interventions [27, 33, 40]. In addition, the nine exergaming studies that examined effects on processing speed or reaction times were all able to show improvements on processing speed.

A progression in ST or DT complexity was provided by nine interventions (cf. Table 3). Four studies used an individualised adaption to the training difficulties (cf. Table 3). All eight studies with an individualised or progressive training that examined executive functions were able to improve these functions. Cognitive-motor training was more effective in comparison to other training interventions if it combined cognitive training with multicomponent exercises including balance or coordination tasks (cf. Table 3).

#### Influence of overall dose

The frequency, session duration and dose of the interventions ranged from 1 to 3 days per week, 20 to 90 min per session and an overall dose of eight to 104 h in total. The shortest intervention duration was 4 weeks and the longest was 16 weeks. The frequency, session duration and dose of the interventions for studies with positive effects on one or more cognitive functions did not differ for studies not showing effects on cognitive function.

To improve global cognitive functioning the total training duration ranged between eight and 104 h [32, 34, 36, 38, 47]. The frequency differed between one or two times per week with 30, 60 or 90 min sessions.

Inhibitory control increased with interventions provided between 12 and 24 h. They were conducted once [41, 42], twice [35] or three times per week [31, 33].

Effects on working memory was shown with training programs that provided 12 up to 36 h of exercise [26, 27, 30, 38, 40, 43, 48]. The frequency differed between once a week [48], twice [30, 38], three times [26, 27, 40] and five sessions [43].

Benefits on set-shifting was gained by exercise interventions with a duration between 12 and 36 h with a frequency of one time per week [29, 38], twice per week [15, 35] or three times per week [27, 33, 40].

The programs that were able to improve visuospatial planning were provided once a week with 60 min for 7 weeks with a total duration of 8 h [36] and three times a week for 20 min with a total duration of 16 h [40].

Attention control increased with exercise interventions of a duration between 12 and 104 h of training [35, 38, 41, 42, 47]. Four of these programs were executed once a week with 60 to 90 min [38, 41, 42, 47]. The intervention by Hiyamazu and colleagues had two 60 min sessions per week.

Improvements of processing speed were found for exercise programs with a duration of eight up to 36 h. Most of these interventions were exergames that were provides three times per week for 20, 30 or 60 min [27, 31, 33, 40, 45]. The other programs had a frequency of two times a week with 30 or 60 min [30, 44, 46] or once session per week with 60 or 90 min [29, 38].

Dual-task costs were reduced with interventions with a duration from twelve provided once a week for 60 min [42] or 60 h of training three times a week with 50 min [25]; MacLean and colleagues [37] did not report their training duration and frequency.

In summary, a dose of at least 60 min training per week (regardless of the number of sessions per week) and a total duration of 12 h of training was necessary to gain positive effects on the different cognitive domains.

## Discussion

The aim of this review and meta-analysis was to investigate whether cognitive-motor interventions improve cognitive function in healthy older people. We focused on interventions that implemented conventional dual-task training and examined whether training methods show a beneficial effect on global cognition and specific executive functions (inhibitory control, working memory and cognitive flexibility). Secondly, we investigated cognitive effects of technology-based dual-task interventions, so called exergames. Overall, we found equal proportions of general cognitive-motor (*n* = 14) and exergame interventions (*n* = 11). Most studies conducted a GDT (*n* = 19), while we also found some studies using SDT (*n* = 6). However, the studies differed in experimental design, training type. Dose as well as outcome or control assessment.

While all studies used valid experimental designs, the training settings differed in many of the investigated trials. Moreover, sample sizes showed great differences. For example, You and colleagues [43], Morita and colleagues [47] and Heiden & Lajoie [46] showed significant results for at least two specific executive functions in their population, with a sample size of less than 20 participants. In comparison, Eggenberger and colleagues [27] included more than 80 participants, which might have led to a higher statistical power and stronger result. These interpretations were supported by the additional sensitivity analysis. After excluding the studies of Morita and colleagues [47] and Eggenberger and colleagues [27] the heterogeneity was reduced, however, the significant results also disappeared. Additionally, the pre-assessment of cognitive ability of participants differed in a majority of studies. Many studies used a valid measurement, e.g. MMSE or MoCA scores, however diversity in testing instruments, e.g. Mini-Cog, 3MS or TDAS score, as well as diversity in the population’s baseline characteristics hamper inter-study comparison. As the reason of this review is to summarise cognitive effects, the same mental baseline condition is of importance to allow a more detailed interpretation of results.

Despite the use of valid assessment instruments to quantify results across all studies, measurements were likewise heterogeneous, hindering a holistic interpretation of effects. For example, studies used different versions of established measurements, e.g. of the Stroop task, which could be delivered as an audio-verbal, as well as visual-verbal or visual-manual task. In general, assessment instruments varied between written or computerised measurements to the active conduction of specific functions, which increased difficulty of comparing assessment structures.

### Effects on global cognition and executive functions

We found that general cognitive-motor interventions improve global cognition and executive functions. All but one study found improvement of at least one specific function. Of note, type of exercise, dose, intervention settings and outcomes differed across trials which allowed for fractional interpretation of the given results only.

#### Effects on global cognition

Our meta-analysis of six studies revealed that cognitive-motor interventions improve global cognition. However, the effect size of this improvement was relatively small (standardised mean difference of 0.6 points on the MMSE and MoCA, 95% CI 0.3–0.9) and the heterogeneity between studies was relatively large (I^2^ = 60%), suggesting that additional evidence is required. Only two studies showed a statistically significant improvement, driving the results of the meta-analyses [27, 47]. Both studies were considered of high quality. The intervention of Morita and colleagues [47] comprised of a total of 104 h of conventional DT training, with 3MMS as their global cognition outcome, and the intervention of Eggenberger and colleagues [27] comprised of 18 h of DT video game training, with MoCA as their global cognition outcome. While the intervention of Eggenberger and colleagues [27] included task-specific exercises with individual adjusted progressions, the program of Morita and colleagues [47] had a training period of 18 months. This suggests that both training specificity and a long intervention period might be beneficial to improve cognitive function.

Nevertheless, it has to be reflected, that the MMSE and MoCA are assessment and screening tools, and not typically used as outcome measures. Therefore, further research is required to identify effective components of cognitive-motor interventions for improving global cognition with more sensitive test batteries (e.g. Mindsteams [49] or CERAD-col [50].

#### Effects on inhibitory control

Six out of eleven DT training programs significantly improved inhibitory control [31, 33, 35, 40–42]. The meta-analysis revealed that cognitive-motor interventions improved inhibitory control. The effect size was Z = 3.66 (*p* < 0.001; mean difference 0.61, 95% CI 0.33–1.09) with high heterogeneity (I^2^ = 95%). Remarkably, this effect was strongest when comparing interventions to active controls, possibly the result of one study by Eggenberger and colleagues [33] with a very large effect size Z = 3.85 (*p* < 0.001; mean difference of 9.0, 95% CI 7.4–10.6). This study comprised of 18 h of DT video game, that included stepping forward, backwards and sideways to music while following cues (i.e. arrows) presented on a screen.

Most programs that showed benefits of inhibitory control included specific training of this cognitive ability in the intervention [31, 33, 41, 42]. The training duration to gain positive effects was at least 12 h. This suggests ageing may affect executive and automatic inhibition differently [51]. Executive inhibition involves consciousness as well as control for the suppression of irrelevant stimuli or responses [51]. These abilities are integrated within an array of training interventions and physical exercise that integrate balance and coordination tasks, because these exercises require skills and cognitive effort to respond to immediate external stimuli that might arise from an unpredictable environment or situations [45]. Therefore, one might argue that these interventions are beneficial to improve executive functions, including inhibitory control. We further found that inhibitory control improved following cognitive-motor exercises that focus on attentional performance. This supports previous findings of improved attentional and inhibition performance following a dual-task training intervention [8, 52–54].

#### Effects on working memory

Working memory only improved in trials which specifically addressed memory abilities during training situations. Most studies used a memory task while conducting physical exercise, which appeared to be stimulating memory function. The results of the meta-analysis for working memory showed no significant effects. A previous review by Kelly and colleagues [55] was able to demonstrate that specific cognitive training can improve the addressed domains of cognition, whereas results of a general mental stimulation were heterogeneous. As indicated by the studies that improved working memory (cf. Table 3), there should be a certain amount of task specific training to gain positive effects on working memory. Accordingly, this might be the reason why the study data remained inconsistent if the task specificity was low.

#### Effects on cognitive flexibility

Set-shifting was analysed as a primary outcome in nine studies (cf. Table 3). Seven of the examined interventions showed improvement of set-shifting after a GDT or a GDT exergaming intervention. Unfortunately, only a few studies could be included in the meta-analysis, showing no significant effects of cognitive-motor interventions on set-shifting. However, set-shifting is an executive function that involves unconscious and conscious cognitive shifting of attention [10, 15]. Such processes can be supported by processing speed. Most of the exergaming interventions (*n* = 9) were able to increase processing speed; future studies could elucidate whether these effects transfer to improvements in set-shifting ability.

### Influence of the training methods

Based on the presented results no major differences in results between methods could be identified. A tendency towards a positive impact on processing speed and attentional control was observed within the exergaming group. On the other hand, general cognitive-motor interventions seemingly have a positive impact on attention, mental flexibility and working memory abilities. Nonetheless these results could not be generalised and were influenced by many methodological factors.

#### Type of intervention

An inter-study comparison between technology-based exercise forms and general dual-task methods was not possible. Types of training methods varied from “off-the-shelf” computerised games to specifically developed modules. For example, five out of fifteen studies used the interactive step-based game “Dance Dance Revolution” (StepMania) out of which three were SDT intervention types. While Schoene and colleagues [31, 40] implemented a home-based setting, the remaining trials chose a supervised laboratory setting. Three other studies used commercially available games for Nintendo Wii or Kinect Xbox while using various equipment, e.g. an additional balance board. Although the use of modern technologies evolved throughout the last decade, a general implementation and usage of exergames still seems unusual in older people. Therefore, the acceptance and feasibility of emerging exergaming modules should be questioned. However, the current findings support the potential of serious gaming and engaging, active and home-based exercise methods for older people [14, 56, 57].

#### Dose of effective interventions

We hypothesised that a higher total intervention dosage would lead to a larger effect size of the intervention. Intervention duration varied between the studies; some trials had a total duration of 6 weeks, while others provided more than 25 weeks of intervention. Trials with a longer total intervention duration and a higher frequency of training sessions seemed to have greater effect size [15, 26–28, 33, 35, 38, 47]. Nevertheless, irrespective of total exercise dose, 24 out of 25 studies showed positive effects on cognitive functions with a training duration of eight to 104 h (cf. Table 1). The frequency differed from one up to three sessions per week, however, the studies with one session per week had a training duration of at least 60 min per session. Based on these findings (cf. Tables 1, 3 and 4), we suggest that a total exercise dose of at least 12 h should be recommended to gain positive effects on EF. Moreover, the duration of all sessions provided per week should be at least 60 min. It has to be noted, that the studies that were not able to improve cognition used the same amount of exercises as shown to be effective to gain positive effects (except of [37] who did not provide information about duration and frequency). Therefore, other reasons such as training specificity or the individual response to cognitive-motor training might have to be considered to explain the results.

#### Specificity of effective interventions

Our hypothesis that the training intervention should be task-specific to gain highest benefits on EF was supported by the available study data. The majority of studies that included task-specific training and testing of EF improved the specific domain of EF. As the meta-analyses revealed inconsistent findings for the different dimensions of EF, further research should assess the effects of different complexities of cognitive tasks on specific executive functions. Several reviews have shown effects of acute and regular exercise on cognition [58, 59]. However, a wide inter-individual variability has been found in the effects of acute and/or regular exercise [60], suggesting there might be responders and non-responders. Therefore, future studies should address aspects of, e.g. responder analysis [61], dose-response relationships [62] and more specific tailoring of the dual-task exercise program [63]. Of note, a large number of general training principles (for an overview cf. Herold et al., 2019 [62]) were not addressed in most studies. There was a lack of detail on addition to training specificity and progression, aspects of variation, overload, reversibility, periodisation, and programming in the provided exercise interventions. These aspects of training methods and mechanisms should be addressed in future clinical trials to allow clinical practice recommendations.

### Limitations

This review has some limitations that need to be addressed. Certain interventions were not included in the review, despite training highly coordinated tasks such as dancing. Following our definition of a DT training, a motor task needed to be performed at the same time of an ongoing observational task, e.g. on a screen. We acknowledge that these types of interventions might add further insights when compared to the more specified DT programs. Second, in order not to unduly exclude studies including people with mild cognitive impairment, we used a slightly lower cut-point (MMSE < 23) to allow subgroup analyses in cognitively healthy versus people with mild cognitive impairment. These different interpretations of non-cognitively impaired older people might have an impact on the study results. Nevertheless, we did not find a systematic direction, if the training interventions gain higher or lower benefits for participants with lower scores in the MMSE.

## Conclusion

Conventional cognitive-motor interventions as well as technology-based exergames can show positive effects on global cognition and inhibition in healthy older people aged 60 and older. The cognitive domains that were influenced by dual-task training programs varied from attentional skills to inhibitory control or mental set-shifting abilities and the reduction of dual-task costs. New technologies can complement home-based and unsupervised training methods, as a useful tool for older people to independently perform adequate training sessions. Of note, results of the meta-analysis were heterogeneous and need to be interpreted carefully due to differences in interventions, measurements and results. Additionally, the influence of different levels of cognitive demands and the influence on specific executive functions should be investigated. While we recommend a minimal dose of 12 h with 60 min per week to achieve effects, this is still preliminary and warrants further research.

## Data Availability

The supporting data is available via the corresponding author.

## Appendix

Search Strategy:

1. Aged or elder* or senior* or geriatric* or older or aged 80) and over mp = title, abstract, heading word, drug trade name, original title, device manufacturer, drug manufacturer, device trade name, keyword, floating subheading word, candidate term word]
2. Dual-task* or exergam* or motor-cognitive or motor$ cogn* or video gam* or computer$ gam*).mp. [mp = title, abstract, heading word, drug trade name, original title, device manufacturer, drug manufacturer, device trade name, keyword, floating subheading word, candidate term word]
3. Intervention or program* or training* or exercise or exercise therapy or physical train*).mp. [mp = title, abstract, heading word, drug trade name, original title, device manufacturer, drug manufacturer, device trade name, keyword, floating subheading word, candidate term word]
4. Cognition or cognitive function* or executive function* or attention*).mp. [mp = title, abstract, heading word, drug trade name, original title, device manufacturer, drug manufacturer, device trade name, keyword, floating subheading word, candidate term word]
